# 

**Published:** 1893-01

**Authors:** 


### CONTENTS.

ORIGINAL ARTICLES—The Radical Cure of Hernia, Particularly with with Children by Thos. H. Manley, M. D., New York ............. 2
Cockleburr in the Air Passage of a Child Successfully Removed by an Intra-Laryngeal Operation; by W. L. Bullard, M.D., Columbus, Ga ............. 11
Extra-Uterine Pregnancy; by Edwin Ricketts Cincinnati, O ....... 13
CORRESPONDENCE—Our London Letter ............. 14
Our New York Letter ............. 12
SOCIETY NOTES—The Clinical Society of Maryland ............. 20
Southern Surgical and Gynecological Association ............. 24
BOOK REVIEWS ............. 33
SELECTIONS AND ABSTRACTS ............. 34–45
The Treatment and Management of Asthma—Subcutaneous Chloral Injection for Puerperal Eclampsia—Carica Papaya in Dyspeptic States—A New Operation for Paralytic Talipes Valgus and the Enunciation of a New Surgical Principle—Scarlet Fever; Its Treatment
EDITORIALS ............. 45
SPECIAL NOTES ............. 50–51
PRESCRIPTION DEPARTMENT ............. 52–53

